# Towards better models for studying human adipocytes *in vitro*

**DOI:** 10.1080/21623945.2022.2104514

**Published:** 2022-07-27

**Authors:** Fabiana Baganha, Ruby Schipper, Carolina E. Hagberg

**Affiliations:** a Division of Cardiovascular Medicine, Department of Medicine Solna, Karolinska Institutet, Stockholm, Sweden; b Center for Molecular Medicine, Karolinska Institutet, Stockholm, Sweden

**Keywords:** Obesity, adipocyte, spheroids, in vitro model, 3D, hypertrophy

## Abstract

With obesity and its comorbidities continuing to rise, we urgently need to improve our understanding of what mechanisms trigger the white adipose tissue to become dysfunctional in response to over-feeding. The recent invent of 3D culturing models has produced several noteworthy protocols for differentiating unilocular adipocytes *in vitro*, promising to revolutionize the obesity research field by providing more representative adipose tissue models for such mechanistic studies. In parallel, these 3D models provide important insights to how profoundly the microenvironment influences adipocyte differentiation and morphology. This commentary highlights some of the most recent 3D models, including *human unilocular vascularized adipocyte spheroids* (HUVASs), developed by our lab. We discuss recent developments in the field, provide further insights to the importance of the microvasculature for adipocyte maturation, and summarize what challenges remain to be solved before we can achieve a culture model that fully recapitulates all aspects of human white adipocyte biology *in vitro*. Taken together, the commentary highlights important recent advances regarding 3D adipocyte culturing and underlines the many advantages these models provide over traditional 2D cultures, with the aim of convincing more laboratories to switch to 3D models.

The prevalence of obesity continues to rise worldwide at an alarming rate, causing a significant increase in obesity-associated diseases such as type 2 diabetes, ischaemic heart disease, stroke, and certain cancers [1,2]. Although recent years have seen the development of several new therapies to potentially help tackle this crisis, including semaglutides which reduce appetite and SLGT2 inhibitors which are both antidiabetic and cardioprotective, we still lack treatments that help over-weight patients maintain metabolic health by directly targeting the adipose tissue [3]. This has led to a renewed interest for studying the human adipose tissue to better understand the underlying mechanisms that promote disease development during weight gain. In particular, the interest of the field has shifted from focusing solely on the degree of obesity, to realizing it is the *functionality* of the adipose tissue that determines much of the risk for disease development [4]. This novel perspective underlines the need for robust and translational experimental systems that would allow adipose tissue function to be studied and understood in more mechanistic detail. However, these systems have not been easy to develop due to the unique characteristics of the lipid-storing white adipocytes, which typically are 40–80 µm in diameter in the lean state and can range up to 200 µm in diameter during obesity [5].

Mature white adipocytes are notoriously hard to study. Unlike most normal cells used for cell culture, adipocytes do not proliferate, they cannot be cryopreserved without breaking, they float and therefore do not attach to common culturing surfaces such as plastic, and they are incompatible with many standard cell biology techniques such as cell counting, flow cytometry, frozen sectioning, or upright microscopy. Some labs therefore use culturing models based on freshly isolated mature adipocytes, such as Membrane Mature Adipocyte Aggregate Cultures (MAAC), but because adipocytes cannot divide, these systems require a constant supply of fresh adipose tissue either from patient surgeries or mouse experiments, something not always readily available [6,7]. This has led the field to traditionally use adipocyte cultures based on adipose-derived progenitors or *pre-adipocytes* instead, which then are differentiated *in vitro* to become lipid-laden adherent cells (for a recent comprehensive overview please see Dufau et al, reference [8]. Using pre-adipocytes has the advantage that these cells can easily be isolated from the stromal vascular fraction (SVF) of the adipose tissue, and more importantly, preserved frozen until utilization. They also attach to plastic, can be expanded at least a couple of passages and are compatible with standard cell biology methods. The differentiated 2D-cultured adipocytes accumulate lipid, respond to lipolytic ques, and express all typical adipogenic markers, albeit at lower levels than their mature counterparts [9]. However, despite numerous attempts to optimize the differentiation protocol, 2D-cultured adipocytes are not mature enough to adapt the typical unilocular morphology seen *in vivo*, characterized by having only a single large central lipid droplet surrounded by a thin layer of cytoplasm and the nucleus squashed to the side. Instead, 2D cultured adipocytes typically retain a multilocular morphology with multiple small (1–5 µm in diameter) lipid droplets per cell, morphologically reminiscent of brown or brite/beige adipocytes despite most cells not showing high thermogenic activity [8]. Despite their wide usage in multiple important publications, we suggest the small size of these lipid droplets is a much more important caveat than generally acknowledged , as the absolute droplet size determines the surface-to-volume ratio of the lipid droplets. It thereby impacts the amount of membrane and membrane-associated proteins synthetized by the adipocytes, as well as the accessibility of the lipolytic machinery to triglycerides and the cells’ lipolytic activity, which is an important measure of adipocyte functionality [10].

In recent years, the invent of 3D culturing methods, which produce more unilocular adipocytes, has therefore sparked an enormous interest in the adipose research community and are on the verge of revolutionizing the way adipocytes are being studied *in vitro*. For example, in the past five years alone, nearly 100 papers that describe the development or usage of ‘*adipocyte spheroids*’ have been registered within PubMed, with most of them describing new variants of 3D culturing protocols. The consensus from these studies is that 3D cultured adipocytes show higher adipogenic gene expression, higher adiponectin secretion and, most importantly, much larger lipid droplets than 2D cultures grown in parallel [8,9,11]. In addition to the wide range of published protocols [12–14], recent publications also include a series of useful methodological papers detailing how 3D cultured adipocytes subsequently can be analysed using for example automated screening platforms, mass spectrometry, tissue clearing or transcripomics, thus opening further possibilities to use 3D cultures to generate new discoveries about basic adipocyte biology [11,15,16]. For example, a recent noteworthy study by the Laushke lab used in-depth multi-omics to identify factors that link 3D culturing to the observed enhanced adipocyte maturation [9]. The study sequenced human pre-adipocytes that were either differentiated and cultured as scaffold-free 3D spheroids or as traditional 2D cultures and compared them to publicly available sequencing data from freshly isolated mature adipocytes. In addition, they also performed lipidomics to characterize differences in lipid content between the models. The study confirms that culturing pre-adipocytes as 3D spheroids leads to adipocytes that more closely resemble the phenotype of freshly-isolated cells both in terms of morphology, lipid droplet size (10–20 µm), expression pattern and lipid composition. Not surprisingly, they identified a more organotypic microenvironment to be the key success factor of 3D models, with parallel 3D and 2D cultures showing significant differences in the expression of extracellular matrix (ECM) components and members of the mechanosensing Hippo pathway, among others. While scaffold-free 3D cultured adipocytes expressed lower levels of ECM proteins in general and shifted their main ECM production from collagen 1 to collagen 4 during differentiation, similar to the changes seen in maturing adipocytes *in vivo*, 2D cultured adipocytes retained a higher, progenitor-like expression pattern of ECM components, characterized by sustained high collagen 1 expression [9]. Such insights should now be used to explore how the adipocyte microenvironment can be further optimized *in vitro*, in order to elucidate how we can push *in vitro* differentiated adipocyte to also grow to the same size as seen *in vivo*.

In line with the above findings, our lab recently published an adipocyte spheroid model where we suggest the presence of vascular sprouts during adipocyte differentiation *in vitro* significantly improves adipocyte maturity and lead to a more unilocular adipocyte morphology after differentiation [17]. The model builds on previous data from the Deschaseaux lab showing that vascularized spheroids can self-organize [18], and on the well-established role of the microvasculature forming the differentiation niche for maturing adipocytes *in vivo*. For example, pre-adipocytes have been shown to reside in close contact with the adipose tissue microvasculature in mice [19], and it has been shown that the post-natal development of mouse fat pads depend on the prior outgrowth of a vascular tree that then guides the subsequent differentiation of white adipocytes [20]. Yet, the importance of vascular sprouts for adipogenic differentiation *in vitro* had remained debated, with some studies showing improved adipogenesis [19,21], while others claiming that the presence of endothelial cells (ECs) could even hamper adipocyte differentiation [22]. The latter might be in part due to the use of human umbilical vein ECs (HUVECs) instead of endogenous, adipose tissue-derived ECs. This is an important factor to consider as vascular beds are highly heterogenous between organs and have organotypic functions and expression patterns, which theoretically could impact adipocyte differentiation [23]. Our optimized Human Unilocular Vascularized Adipocyte Spheroid (HUVAS) model uses human SVF cells, which contain both pre-adipocytes and ECs, culturing them first in endothelial growth media to promote EC survival and proliferation, and then imbedding the spheroids in growth factor-reduced (GFR-) Matrigel, which together stimulates the outgrowth of vascular sprouts prior to adipogenic differentiation [17]. When compared to *in vitro* differentiated adipocytes from the same cell donors, cultured either in 2D or as scaffold-free 3D spheroids, HUVAS show enhanced differentiation of adipocytes with a more unilocular morphology and the largest average lipid droplet sizes that we have seen to date (with average lipid droplet sizes between 20–40 µm). Moreover, we could demonstrate that HUVAS are functionally responsive to lipolytic and insulin stimulations and become enlarged (hypertrophic) when fattened with Intralipid, a triglyceride-rich lipid mixture. Continuing to explore the importance of the microvasculature for adipocyte morphology and the success of the model, we have since this publication continued to characterize HUVAS devoid of vascular sprouts, generated by omitting vascular growth factors from the culture media and further inhibiting angiogenesis by neutralizing the major angiogenic inducer Vascular Endothelial Growth Factor A (VEGF-A) in the media (as described in reference 17). The resulting spheroids are not only significantly smaller (as shown in our original publication) but also display predominantly multilocular adipocytes containing smaller lipid droplets and secreting lower levels of adiponectin to the media, showing that development of unilocular adipocytes is at least in part dependent on the presence of endothelial sprouts within the adipocyte growth niche (Figure 1). Note that the reduced adiponectin secretion may result from a lower number of differentiated adipocytes, or from less well differentiated cells, as the results are shown as concentration per well and not normalized to the number of differentiated adipocytes in each well. Further studies are thus needed to understand the mechanisms by which the vasculature affects adipocyte differentiation and/or maturation, in order to discriminate between direct effects from the absence of vasculature, and secondary effects brought upon by the subsequent reduction in adipogenic potential. Notably, these results mimic those seen *in vivo* when VEGF-A signalling was inhibited in mice during fat pad development, which similarly led to the formation of only multilocular fat cells with smaller lipid droplets [20]. HUVAS therefore provides a unique model system to explore the underlying molecular mechanisms of endothelial-adipocyte crosstalk in more detail.

**Figure 1.**
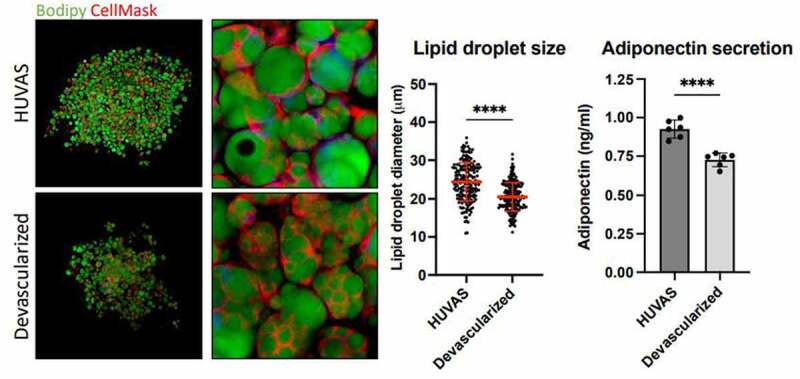
Devascularized HUVAS display less mature adipocytes. HUVAS were devascularized prior to differentiation as described in the original paper [17]. Subsequent confocal imaging of spheroids stained with Bodipy (green, to detect lipid droplets) and CellMask (detecting both lipid droplet and plasma membranes) showed widespread multilocularity. In the middle, quantification of the lipid droplet sizes for control (n = 2) and desvacularized (n = 3) spheroids is shown. Each point represents one lipid droplet/cell, with 200 lipid droplets quantified for each spheroid, and the median with standard deviations shown as a line. Statistics were calculated using two-way ANOVA and Graph Pad Prism 9. To the right, Adiponectin secretion to the media is shown, which was also reduced (detected for n = 6 spheroids per condition as described in reference 17).

One caveat of HUVAS is the use of GFR-Matrigel as scaffold because of its undefined composition, mouse oncogenic origin and the occurrence of slight variations between Matrigel batches. GFR-Matrigel is reported to consist of about 60% laminins, 30% collagen 4 and smaller amounts of other ECM constitutes such as entactin, heparan sulphate proteoglycans and fibronectin [24]. The high level of collagen 4, which is one of the main constituents of the ECM of mature adipocytes [9], might be one of the factors contributing to its suitability for adipocyte cultures. When comparing several other xeno-derived or synthetically synthesized gels available on the market, we have to date not found a single one that could fully substitute GFR-Matrigel in terms of sprouting efficiency and adipocyte morphology, provoking the question if it is solely collagen 4 or also other gel components that contribute to the success of the GFR-Matrigel (Figure 2). Our data furthermore shows that the stiffness (concentration) of a particular scaffold greatly influences its suitability for adipocyte cultures, suggesting GFR-Matrigel might simply provide an optimal combination of ECM stiffness and composition, enhancing both vascular sprouting and subsequent adipogenic differentiation. Interestingly, numerous reports, including data from our lab, suggest the adipose tissue of lean individuals to be more collagen-dense than that of individuals with obesity [25]. Development of obesity also impairs adipogenesis and leads to vascular rarefaction, both contributing to development of adipose tissue dysfunction [4,26]. This makes it attractive to hypothesize that the decreased densities of both the ECM and the vasculature during obesity together could be deteriorating the growth niche of pre-adipocytes, and thereby lowering the adipogenic capacity of the obese subcutaneous adipose tissue. However further research is needed to test this hypothesis. Notably, most in vitro models either lack vascular sprouts and/or have very different ECM composition as compared to mature adipocytes [9], making them suboptimal for these studies and further highlighting some of the advantages with our HUVAS model. In support, our lab recently contributed to a paper showing the positive effects of lipoxins on adipose tissue, where *in vitro* results from cultured mature adipocytes, HUVAS and 2D cultures are compared, concluding that whereas HUVAS respond similarly as the mature adipocytes to lipoxin treatment, 2D-differentiated adipocytes remained unresponsive [27]. These results again highlight the importance of using representative cell culture models, and suggests we still have a lot to learn about how to optimally differentiate human adipocytes in a culturing dish. Further research into the crosstalk between vascular sprouts and pre-adipocytes, as well as the optimal composition of the ECM during adipogenic differentiation is clearly needed, and such insights would help enhance future culture models and clarify if the adipogenic capacity at least in part is diminished during obesity due to structural changes in adipocyte microenvironment.

**Figure 2.**
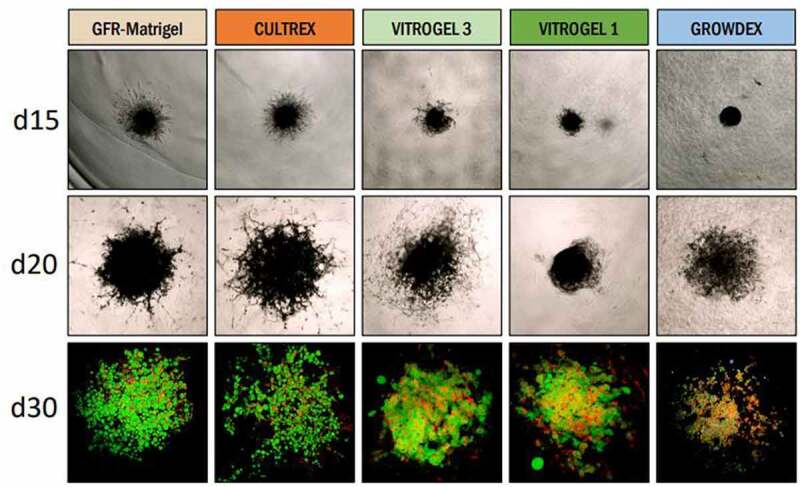
Impact of scaffold on vascular sprouting and adipocyte differentiation. HUVAS were cultured as previously described [17] and imbedded on day 6 of culture either in GFR-Matrigel (Corning), GFR-Cultrex (RnD Systems), VitroGel 1 or 3 (Tebu-Bio), or in GrowDex (UPM Biomedicals), and adipocyte differentiation was initiated four days later. Light microscope images were taken to see sprouting development (row 1 and 2). Note the differences in vascular sprouting visible on day 15 (top row) and the subsequent variation in adipocyte differentiation, as judged by confocal images of spheroids stained with Bodipy (bottom row, green, detecting lipid droplets) and CellMask (red, detecting both lipid droplet and plasma membranes).

Lastly, our paper describing the HUVAS model also includes a protocol to fatten the spheroids, inducing adipocyte hypertrophy and subsequent development of adipocyte dysfunction, thus allowing us to mimic the detrimental effects of weight gain *in vitro* [17]. The protocol uses Intralipid, a soybean oil-based lipid mixture consisting of mostly unsaturated triglycerides, phospholipids and glycerine. It is routinely used in the clinic as neonatal lipid supplement, during fertility treatments and to prevent drug-induced myocardial damage in patients. Importantly, Intralipid is, in contrast to most other fattening protocols used by the adipose field, *anti-inflammatory* instead of pro-inflammatory, and thus serves not to induce insulin resistance directly but only to enlarge the adipocytes and thereby recapitulating the early effects of over-nutrition. It should be mentioned that even short-term high fat feeding has been shown to provoke an inflammatory response in mouse adipose tissue, indicating that over-nutrition *per se* not only enlarges adipocytes but also may impact their inflammatory milieu [28]. By fattening HUVAS with triglycerides, we hope to have a model that more closely can mimic the initiating disease mechanisms coupled to weight gain and thereby could increase our understanding of how adipocyte function gradually is altered upon obesity. Nevertheless, a major caveat of the HUVAS model remains the absence of tissue-resident macrophages and other immune cells, that to our knowledge to date only have been detected within one 3D adipose tissue culture model [29]. The successful addition of immune cells to all 3D culture models therefore should be the next major goal for the research field, as it is only when all major cellular components are present that these models start to fully resemble the *in vivo* adipose tissue and mimic its responses to chronic inflammation, lipid accumulation and insulin resistance.

An advantage of the spheroid format is that it allows us to control many important aspects of adipocyte differentiation and growth. Firstly, the cell number is the same between wells (=spheroids), omitting the often-difficult problem of how to normalize data between adipocyte populations where the cells often have different size, protein content and DNA content [7]. Inter-well variability can be further reduced by ensuring SVF cells are well mixed, by reducing evaporation and evenly embedding the spheroids in Matrigel. For example, to reduce evaporation, cells are only plated in the inner wells of the 96-well plates while the borders are instead filled with only PBS. In addition, the culturing plate can be stored in the incubator between two additional plates filled entirely with PBS, adding moisture and pressure from the top and thus reducing evaporation. When spheroids are imbedded in GFR-Matrigel, the media volume in the wells is checked to be even between wells, assuring even polymerization and thus vascular development among wells. These extra control steps help avoid differences in the growth niche and thus keep the constant cell number between samples. Secondly, the spheroid format allows fattening of a subset of spheroids from each donor, and using unfattened spheroids from the same donor as controls, thus enabling the separation between (epi)genetic variability and direct effects of the lipid treatment. However, we have noticed that similarly to the clinic, Intralipid tends to become oxidized with time and thereby less effective, and therefore preferentially should be stored frozen in aliquots until usage. We also believe its effectiveness is in part due to the mixture being an emulsion, and we are currently trying to understand what molecular machinery is required by the adipocytes for the uptake of Intralipid-derived fatty acids, as well as which of its components are required for significant adipocyte fattening to occur.

Taken together, despite many recent advances in the field of 3D adipocyte culturing, some key challenges remain. These include the difficulty of obtaining *in vitro*-differentiated adipocytes fully mirroring the *in vivo* phenotype, with a solely unilocular morphology and lipid droplet sizes in the range of that seen in humans *in vivo* (60–120 µm). In addition, most published 3D models except one model published by the Weber lab lack significant contributions of immune cells, something that would help to fully recapitulate human adipose tissue phenotype [29]. As our insights change, the adipose field should also reassess which *in vitro* characteristics define a fully differentiated adipocyte; is some lipid content and the expression of traditional adipogenic genes such as *PPARG, FABP4* and *PLIN1* enough to claim full adipogenic differentiation, or are their adipokine secretion, morphology, or total lipid content equal or even more important characteristics? We predict that the evolution of improved *in vitro* adipocyte models may with time force us to redefine which cultured cells truly mature to become adipocytes. One should also be careful when using Bodipy to evaluate the lipid content of lipid-poor cells as the dye will readily stain cell membranes if added in excess to the total lipid content. This was previously shown by us using flow cytometry, and we showed that Lipidtox is instead a more specific dye to use when staining cells with low lipid content and/or multiple small lipid droplets [30]. By building on the many interesting emerging studies, such as the characterization of the ECM of mature adipocytes and acknowledgement that the vasculature actively contributes to the differentiation of adipocytes [17,31], we believe the adipose field is on the right path towards developing models that in the nearby future will allow us to study the human white adipose tissue *in vitro* more accurately.

## Data Availability

The data that support the findings of this study are available from the corresponding author, CEH, upon reasonable request.
